# Open defecation practice among households with latrines in rural communities of Ararso District, Somali Region, Eastern Ethiopia

**DOI:** 10.3389/fpubh.2024.1394351

**Published:** 2024-05-01

**Authors:** Abdifatah Mohamud Ismail, Musse Ahmed Ibrahim, Mahammoud Mohammed Abdi, Abraham Geremew, Yohannes Mulugeta, Dinku Mekbib Ayele, Abera Cheru

**Affiliations:** ^1^Somali Regional State Health Bureau, Jigjiga, Somali Region, Eastern Ethiopia; ^2^School of Environmental Health Science, College of Health and Medical Sciences, Haramaya University, Harar, Ethiopia

**Keywords:** open defecation, households with latrines, Ararso District, taboo, Somali Region

## Abstract

**Background:**

Open defecation contributes to the spread of different feco-oral diseases. Therefore, access to a latrine is strongly recommended, as it considerably reduces the risks. Even though provision of latrine facilities alone does not guarantee the desired health benefits, they should be integrated with behavior change. In Ethiopia, efforts have been made to increase the coverage of latrine facilities. However, evidence on how consistently households use it is limited. Most prior studies focused on latrine utilization among households, and limited evidence is available about open defecation practices among households with latrines and associated factors. Thus, this study is critical for developing effective intervention approaches to prevent open defecation among households with latrines.

**Objective:**

The aim of this study was to assess the open defecation practice and associated factors among households with latrines in rural communities of Ararso District, Somali Region, Eastern Ethiopia, 2023.

**Method:**

A community-based, cross-sectional study design was employed among households with latrines in the district. A total of 632 households latrines were selected using a systematic sampling technique. Data were collected using a structured questionnaire and an observational checklist. The questionnaire was designed in KoboTool box, Humanitarian Response software, and the data were collected using the Kobo Collect version 2023.2.4 mobile application. The data were downloaded from the server in the Microsoft Excel format for data cleaning before being exported to STATA version 14 for analysis. Bivariate and multivariable analyses were employed to investigate the relationship between outcome and independent variables. Odd ratios with 95% confidence intervals were utilized to assess the association between the outcome and the predictor variables. A P-value of <0.05 was used as the threshold point for statistical significance.

**Result:**

In this study, the prevalence of open defecation practice among households with latrines was 32.4% (95% CI: 28.1, 35.9). Sex of the household (AOR = 1.60, 95% CI: 1.06, 2.4), educational status (AOR = 2.40, 95% CI: 1.08, 5.53), family size (AOR = 1.62, 95% CI: 1.22, 2.78), the presence of under-5-year-old children in the house (AOR = 1.84, 95% CI: 1.19, 2.75), the need for latrine maintenance (AOR = 2.37.95% CI: 1.62, 3.48), current status of the latrine (AOR = 2.37, 95% CI: 1.62, 3.48), and latrine cleanness status (being unclean) (AOR = 1.91, 95% CI: 1.29, 2.81) were significantly associated with open defecation practice among households with latrine.

**Conclusion:**

The study concluded that open defecation was significantly practiced by households with latrines. This revealed that the presence of a latrine alone was insufficient to considerably reduce open defecation. To alleviate this problem, the government and health workers, in collaboration with the health bureau, should promote frequent sanitation and hygiene education in the communities.

## Introduction

Open defecation (OD) is the act of defecating in fields, forests, bushes, bodies of water, or other public areas without properly disposing of human waste (1). There can be different reasons for open defecation being practiced; it could be a voluntary, semi-voluntary, or involuntary choice (2). Most of the time, a lack of access to a toilet is considered the main reason. However, in some places, even people with toilets in their homes could prefer to defecate in the open area due to various factors, including poor construction and management of facilities, family size, educational status of owners, the presence of children, shortage of water supply, cleanliness of the toilet, personal preference, cultural taboos, and others (3–6).

Previous studies provide evidence and insights into anthropological-psychological factors like cultural taboos, cultural beliefs, gender dynamics, cleanliness perceptions, and lack of awareness that could hinder latrine utilization and underscore the importance of considering these perspectives in sanitation interventions and behavior change campaigns. For instance, a study by Sinha et al. in Odisha, India, highlighted the impact of taboos on latrine adoption. The researchers found that certain beliefs, such as the fear of evil spirits or ancestral deities being offended, hindered latrine utilization (7). Financial resources available in the community can enable households to invest in the maintenance and repair of their latrines, facilitate the upgrading of latrine facilities, such as adding handwashing stations or improving ventilation, which can enhance comfort and convenience, thereby promoting utilization (8).

Studies conducted in different parts of India showed that the level of open defecation practice among households with latrines was 54.8% in Dharmapuri District, 15% in Hubballi and Dharwad, and 45% in rural north India (4–6). In Ethiopia, the level of open defecation varies, with 16.9% in Wondo Genet District (9) and 27.8% in Machakle District, Northwest Ethiopia (2). A human excreta contains a large number of germs. When humans defecate in the open, flies feed on the waste and can carry small amounts of the excreta away on their bodies and feet, which causes contamination of the environment and the propagation of flies, which in turn causes the spread of diseases (8).

According to WHO estimates, 1.8 million people in low- and middle-income countries have severe trachoma (9), a primary cause of vision impairment that is carried by flies that spawn on human excrement and have a propensity to spread through an infected person's ocular discharge. The OD practice aids in the transmission of microorganisms that cause diarrheal diseases, and children are the most vulnerable (10). Globally, an estimated 2 billion cases of diarrhea occur each year, and 1.9 million children under the age of 5 years, mostly in developing countries, die from diarrhea (11). In Ethiopia in particular, diarrheal diseases alone accounted for 23% of the causes of child mortality (10). Moreover, OD can have social and cultural implications, including a loss of dignity and privacy. It can disproportionately affect women and girls, who face safety risks and reduced access to sanitation facilities, leading to compromised menstrual hygiene management and increased vulnerability to gender-based violence (12).

The presence of a latrine alone is insufficient to considerably reduce open defecation. To accomplish the process, people's behaviors at this contextual and individual level might need to change significantly (13). Studies in rural India revealed that households perceived open defecation as more convenient and time-saving if located far from their homes (14). Other studies in rural India identified that households practiced open defecation when latrines were overcrowded, unclean, or lacked privacy, indicating the influence of inadequate sanitation infrastructure (15). A previous study conducted in Ethiopia also showed that not attending formal education, having more than five family members, the presence of under-5-year-old children, preferring leaves as anal cleaning material, and having a latrine that needs maintenance influenced a household latrine and led to open defecation practices despite having a latrine (3).

Both governmental and non-governmental organizations (NGOs) have worked to build latrines and use them to achieve open defecation-free status using a variety of strategies, including the introduction of market-based sanitation (MBS) and community-led total sanitation and hygiene (CLTSH). The majority of the households (HHs) in the study site adopted these approaches. However, achieving and maintaining the status of open defecation-free was still challenging.

Even though plenty of studies were conducted on open defecation practices in general, the information on open defecation practices among households with latrine was scarce. To effectively create strategies for interventions and to completely eradicate open defecation, it is imperative to understand the factors that contribute to this paradoxical habit. Therefore, this study was aimed at assessing the open defecation practice and identifying the factors that contribute to the practice among households with a latrine in Ararso woreda, Jarar Zone, Somali region, Eastern Ethiopia.

## Materials and methods

### Study design, area, and period

A community-based cross-sectional study was conducted among households with latrines in rural communities of Ararso District, Jarar Zone, Somali Region, which is located in eastern Ethiopia, at 713 km east of Addis Ababa (the capital city of Ethiopia). The district's overall population is 143,516, with 33,376 households. The district is divided into 17 administrative units (kebeles), of which five are urban and 12 are rural. In the district, there are 4 health centers and 10 health posts. The latrine coverage in the district was 31% (District Health Bureau annual report). The study was conducted from 10 to 27 September 2023.

### Populations

All rural households with latrines in Ararso District were the source population, whereas rural households with latrines in the selected kebeles of the rural community of Ararso District during the study period were the study population.

### Sample size determination

The final sample of 632 households with latrines was estimated using the single population proportion formula by considering a 95% confidence level, 5% margin of error (d), 27.8% population proportion of open defecation, which was taken from a study conducted in Machakl District, East Gojjam Zone (3), a design effect of 2, and a 5% non-response rate.

### Sampling technique and procedures

**Figure 1:**
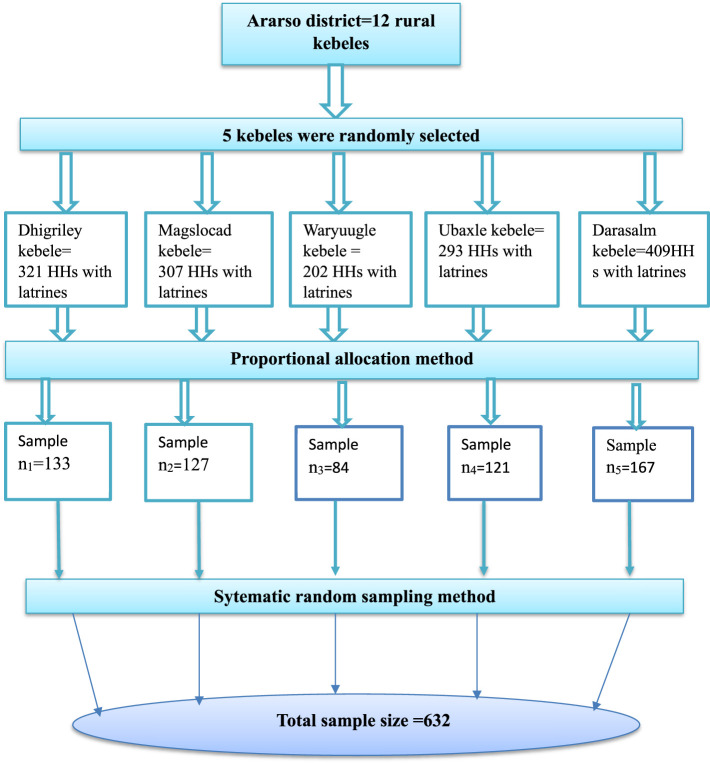
Schematic presentation of sampling procedure in Ararso District, Jarar Zone, Somali Region, Eastern Ethiopia, 2023.

The sample was selected using a multistage sampling method. Five Kebeles were chosen from the district using a simple random sampling method. The sample size was proportionally allocated based on the number of households with latrines existing in each kebele. Finally, a systemic random sampling technique was employed with a K^th^ value of 2 to select households from selected kebeles (Figure 1).

### Data collection instruments and methods

The data were collected using a structured questionnaire that was adopted after reviewing previous studies (3, 16, 17) and an observational checklist. The questionnaire was first prepared in English, translated into Somali language, and finally translated back into English by an expert who was fluent in both languages to maintain its consistency. The questionnaire was designed on the KoboToolbox—Humanitarian Response software platform (https://eu.kobotoolbox.org) and linked to the Kobo Collect version 2023.2.4 mobile application using the server URL (https://kc-eu.kobotoolbox.org), user name, and password for data collection. The data were collected using interviews and observation. Five college graduate professionals who had data collection experience using the Kobo Collect mobile application were involved in data collection. Two B.Sc. environmental health professionals were involved in supervising the data collection process.

### Study variables

The Open defecation practice was the outcome variable of this study, whereas **socio-demographic factors** (family size, gender, household educational status, marital status, occupational of household head, and the presence of students in the households); **environmental or latrine-related factors** (latrine type, service year of toilet, distance of toilet, hygienic condition of toilet, frequency of latrine cleaning, superstructure of latrine, and the availability of bushes, forests, beaches, and open spaces around the house); **knowledge and attitude-related factors** (personal attitude on open defecation practice, initiation of toilet use, knowledge on the effect of open defecation, and knowledge on the benefits of latrine use), and **other variables** (community norm, traditional belief, and taboo) were independent variables of this study.

### Operational definition

#### Open defecation practice

In this study, open defecation practice was considered to be any household with latrines and at least one family member defecating either in fields, forests, bushes, open bodies of water, lakes, ponds, or other open spaces (17).

#### Knowledge on open defecation practice

The response to knowledge questions about open defecation (OD) practice was summed up, and a total score was computed from 10 questions related to the hygiene effect on open defecation (OD). The respondents were considered to have good knowledge if they answered greater than or equal to the mean score (6.28) (6).

#### Attitude toward open defecation practice

Individual attitudes toward open defecation were determined by 12 attitude questions using the Likert scale. All of the individual responses were added together to provide the score, and those who answered above the mean indicated a positive attitude toward open defecation (OD) practice, while those below or equal to the mean (36.7) indicated a negative attitude toward open defecation (OD) practice (3).

#### Traditional beliefs

People's beliefs about latrine use, either negative or positive, six questions will be summed and mean (9.9) and above will be considered as having positive traditional beliefs otherwise negative (18).

#### Taboos

We considered open defecation taboo if the participants answered more than the mean (7.79) of the five questions asked; otherwise, it is not taboo (18).

### Data quality control

A pretest was carried out on 5% of the selected households who had latrines in two kebeles in Degahbour Woreda other than the study area, which had similar socio-economic characteristics to the selected kebeles, to check its clarity before the actual data collection was carried out. The training was given to data collectors and supervisors for 2 days before actual data collection took place. The training was focused on how to fill out the questionnaire and how to approach the respondents. Supervisors performed close site supervision during the whole data collection period. The collected data were checked for completeness, consistency, accuracy, and clarity daily by the supervisors and principal investigator on the data server, and feedback was given for data collection throughout the progress of data collection.

### Data analysis

The data were downloaded from the KoboTool Box—Humanitarian Response software server URL (https://kc-eu.kobotoolbox.org) in the Microsoft Excel format. Then, the data were cleaned, checked for its completeness, and exported to STATA version 14 for data analysis. Descriptive analyses like frequency distributions and percentages for categorical variables and mean and standard deviation for normally distributed continuous variables were used to describe the characteristics of variables. The analyzed results were presented using texts, tables, charts, and graphs. Both binary logistic regression analysis and multivariable logistic regression analysis were performed. In binary regression analysis, factors with a *p* < 0.25 were considered to have an association with the outcome variable. Then all variables that showed an association in binary logistic regression analysis at (*p* < 0.25) were considered for multivariate analysis to control all possible confounders and identify predictors of open defecation practice among households with latrines. Multicollinearity was checked with variance inflation factors (VIF). In multivariate logistic regression analysis, a *p* < 0.05 was used to declare statistical significance. An adjusted odd ratio along with a 95% CI was used to show the strength of the association between open defecation practice and associated factors. Model fitness was checked using the Hosmer-Lemeshow goodness-of-fit test.

### Ethical consideration

To undertake this study, the Institutional Health Research Ethics Review Committee (IHRERC) of the College of Health and Medical Sciences, Haramaya University, provided ethical approval. A formal letter was submitted to the Ararso Wereda Health Bureau, and authorization was obtained to perform this study. The purpose of the study was clearly explained to participants, and all information acquired from them was kept in secrecy. Inform the participants that they are free to answer questions and end the interview at any time. Finally, the participants provided informed consent for their participation in the study.

## Results

### Socio-demographic characteristics of households

A total of 630 households from five kebeles in the Ararso District were included in the study, resulting in a response rate of 99.7%. Out of these, 433 (69%) household heads were female. The mean age of the HH heads was 35.62 years, with a standard deviation (±SD) of 10.34 years. Regarding the marital status of the HH heads, 490 (78%) were married. Of these, 327 (52%) of the HH heads were wives. Nearly half, 322 (51%) of the households, had a family size greater than six, whereas about 401 (64%) of the HHs had under-5-year-old children. The majority, 459 (73%) HHs, had school-age children (Table 1).

**Table 1:** Socio-demographic characteristics of respondents in Ararso District, Jarar Zone, Somali Region, Eastern Ethiopia, 2023 (*n* = 630).

**Variable**	**Category**	**Frequency**	**Percentage**
Household head sex	Male	197	31
	Female	433	69
Household head age (years)	15–29	209	33.2
	30–44	282	44.8
	≥44	139	22.1
Household head marital status	Married	490	77.8
	Single	35	5.6
	Widowed	30	4.8
	Divorced/ separated	53	11.9
Household head educational status	Illiterate	185	29.4
	Primary school (1–8)	310	49.2
	Secondary school (9–12)	47	7.5
	Diploma and above	139	22.06
Household head occupation status	House wife	327	51.9
	Farmer	139	22.1
	Merchant	43	6.8
	Daily laborer	58	9.2
	Government employee	28	4.4
	Self-employee	35	5.6
Family size	≤ 6	308	48.9
	>6	322	51.1
The presence of an under-5-year-old child in the house	Yes	401	63.7
	No	229	36.3
The presence of school-age children	Yes	459	72.9
	No	171	27.1

### Open defecation practices among households with latrine

**Figure 2:**
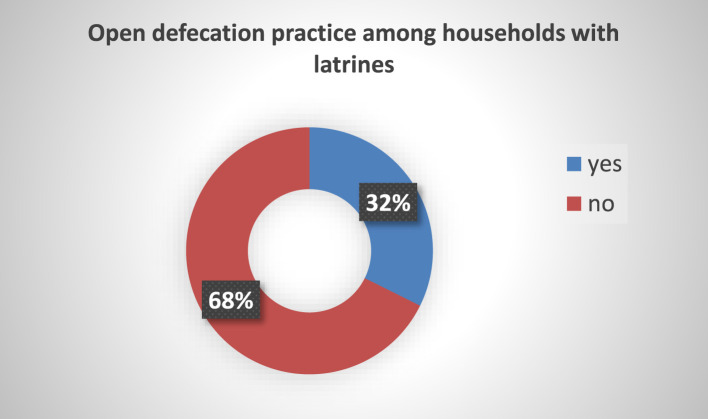
Open defecation practice of HHs with latrines in Ararso District, Jarar Zone, Somali Region, Eastern Ethiopia, 2023 (*n* = 630).

Out of the 630 households (HHs) included in the study, 204 (32.4%) reported practicing open defecation (Figure 2).

Among the participants who practiced OD, 67 (32.8%) respondents stated that they defecated in the nearest bushes or open spaces around the house. Of these, 31.9% of households always practiced open defecation. Among those practicing OD, 41 (30.9%) mentioned the large squat hole of the latrine, and 81 (39.5%) stated the offensive odor from the latrine as a factor that pushed them to defecate outdoors. Also, among those practicing OD, 58 (28.4) were under-5-year-old children. Regarding households with babies, 322 (51%) households disposed of their baby's feces into latrine using Popo (Table 2).

**Table 2:** Open defecation practice and child feces disposal practice among family members in Ararso District, Jarar Zone, Somali Region, Eastern Ethiopia, 2023 (*n* = 204).

**Variables**	**Variable category**	**Frequency**	**Percentage**
Place of practice for OD	In the bushes around the house	67	32.8
	Defecate on open field	82	40.2
	Backyard	55	27.0
OD practice frequency	Always	65	31.9
	Mostly	47	23.0
	Sometimes	43	21.1
	Rarely	49	24.0
Reasons for practicing OD	Big squat hole of latrine	63	30.9
	Offensive odor	81	39.7
	Latrine structure is not safe	34	16.7
	Slab is not safe to defecate	26	12.7
Who practices OD among family members?	Husband/wife	32	15.7
	Above-5–year-old children	71	34.8
	Under-5-year-old children	58	28.4
	Ill person/pregnant	43	21.1
Baby's feces disposal practice	Cover with soil or stone	32	15.7
	Put into the latrine using Popo	322	51.1
	Put into drain/ditch	46	7.3
	Thrown with garbage	140	22.2
	Left open	122	19.4

### Knowledge of respondents on open defecation practice

Out of 630 participants, 395 (62.7%) respondents reported that defecating in any place had health problems, and 357 (56.7%) respondents knew that human feces were the main cause of diarrhea. Notably, 360 (57.1%) respondents said that poor latrine conditions encouraged open defecation, and 406 (64.4%) reported that household latrine improved personal hygiene. In total, 398 (63.2%) reflected that latrine had an effect on increasing overall family health and breaking the chain of disease transmission, and 249 (39.5%) of the respondents reported that handwashing with soap and water could prevent diarrheal disease. Regarding the respondents' knowledge of OD, 354 (56.2%) of the study subjects had poor knowledge (Table 3).

**Table 3:** Knowledge of respondents on open defecation practice among households with latrines in Ararso District, Jarar Zone, Somali Region, Eastern Ethiopia, 2023 (*n* = 630).

**Variables**	**Category**	**Frequency**	**Percentage**
Defecating at any place has health problems	Yes	395	62.7
	No	235	37.3
Human feces are the main cause of diarrheal disease	Yes	357	56.7
	No	273	43.3
There is a risk of getting diarrhea if a neighbor practices open defecation	Yes	392	62.2
	No	238	37.8
Poor latrine conditions encourages open defecation practices	Yes	360	57.1
	No	270	42.9
The presence of flies in the latrine encourages open defecation	Yes	392	62.2
	No	238	37.8
Latrine improves personal hygiene	Yes	406	64.4
	No	224	35.6
Latrine utilization breaks the chain of diarrheal disease transmission	Yes	398	63.2
	No	232	36.8
Checking the condition of the latrine on a regular basis is not important	Yes	252	40.0
	No	378	60.0
Children are remarkably more vulnerable to diarrhea than adults	Yes	391	62.1
	No	239	37.9
Handwashing practices with water and soap prevent diarrheal disease	Yes	249	39.5
	No	381	60.5
Overall respondents' knowledge on open defecation practice	Poor knowledge	354	56.2
	Good knowledge	276	43.8

### Attitudes of respondents toward open defecation practice

In total, 281 (44.6%) respondents strongly believe that the presence of feces all over the floor of the latrine forced users to opt for the practice of OD, whereas 172 (27.3%) respondents thought that it was embarrassing when people could see others defecating in the open. Notably, 240 (38.1%) respondents disagreed that defecating on the pond or in the river was not a problem. In addition, 143 (22.7%) respondents disagreed that the majority of illnesses were caused by OD practice, while 196 (31.1%) respondents agreed that punishment for OD helps all households discontinue the practice. Almost half, 324 respondents (51.4%), had a positive attitude toward OD practice, while 306 (48.6%) had a negative attitude (Table 4).

**Table 4:** Attitude of respondents on open defecation practice in Ararso district, Jarar Zone, Somali Region, Eastern Ethiopia, 2023 (*n* = 630).

**Variables**	**Strongly disagree**	**Disagree**	**Neutral**	**Agree**	**Strongly agre**
The presence of feces all over the floor of the latrine forces the users to opt for the practice of OD	281 (44.6)	74 (11.7)	12 (1.9)	122 (19.4)	141 (22.4)
Unsafe practices should be discouraged	23 (3.7)	83 (13.2)	101 (16.0)	189 (30.0)	234 (37.1)
Sharing a latrine between HHs may lead to poor latrine condition, which eventually Discourage	134 (21.3)	179 (28.4)	51 (8.1)	165 (26.2)	101 (16.0)
It is embarrassing when people can see others defecating in the open field	104 (16.5)	177 (27.9)	75 (11.8)	172 (27.3)	102 (16.2)
It is not a problem to defecate on the pond or in the river	150 (23.8)	240 (38.1)	68 (10.8)	62 (9.84)	110 (17.46)
Human excreta smells bad and attracts many flies inside the latrine facility, so defecating in the bush is more comfortable	145 (23.2)	158 (25.1)	41 (6.5)	188 (29.8)	98 (15.6)
Feel uncomfortable when using public toilets and have a health problem	109 (17.2)	140 (22.1)	15 (2.4)	225 (35.7)	141 (22.4)
Diseases will spread to children if family members share the latrine	91 (14.4)	126 (20.0)	68 (10.8)	192 (30.5)	153 (24.3)
Children's feces are not harmful, and defecating in open spaces by children is common	147 (23.33)	161 (25.56)	64 (10.2)	146 (23.17)	112 (17.8)
People who defecate in the open put the entire community at risk of disease	103 (16.3)	112 (17.8)	82 (13.2)	179 (28.4)	154 (24)
Most of the illnesses occur as a result of OD practice	106 (16.8)	143 (22.7)	58 (9.1)	178 (28.3)	145 (23.0)
Punishment regarding OD helps all households end the practice	106 (16.8)	177 (28)	40 (6.3)	196 (31.1)	111 (17.6)
Overall attitude of respondents	Positive attitude			Negative attitude	
	324 (51.4%)			306 (48.6%)	

### Socio-cultural, behavioral characteristics, and traditional belief questions

Among 630 respondents, 428 (67.9%) stated that the practice of OD did not violate their tradition, and 466 (74%) stated that there were no punishments associated with OD in their village. Notably, 411 respondents (65.2%) preferred to defecate at night. Furthermore, 429 (68.1%) of respondents stated that OD practice did not provide manure for agricultural activities. In addition, 453 (71.9%) respondents said that no one objected when someone practiced OD. Generally, 403 (64%) had a positive traditional belief about open defecation practice, whereas 227 (36%) had a negative traditional belief (Table 5).

**Table 5:** Socio-cultural characteristics of respondents on OD practice in Ararso District, Jarar Zone, Somali Region, Eastern Ethiopia, 2023 (*n* = 630).

**Variables**	**Category**	**Frequency**	**Percentage**
OD practice is a part of community tradition	Yes	202	32.1
	No	428	67.9
There is a norm in place that advises or discourages when someone practices open defecation	Yes	164	26.0
	No	466	74.0
People in the community object when a person defecates in public	Yes	177	28.1
	No	453	71.9
Women mostly defecate at night	Yes	411	65.2
	No	219	34.8
Open defecation is a continuation of an ancestor's way of life	Yes	179	28.4
	No	451	71.6
Defecating in agricultural fields/gardens provides manure	Yes	201	31.9
	No	429	68.1
Overall traditional beliefs of respondents	Negative	227	36.0
	Positive	403	64.0

### Socio-cultural and behavioral characteristics taboo-related questions

Of the 630 respondents, 277 (36%) stated that there was a taboo for defecating in an enclosed space (toilet). On the contrary, 229 (36.3%) respondents claimed that menstruating girls defecating in the toilets used by family members were forbidden. Similarly, 345 (54.8%) respondents claimed there was no taboo in sharing a latrine with a mother-in-law or father-in-law, 352 (55.9%) said there was no taboo associated with using a latrine with a son-in-law or daughter-in-law, and 335 (53.2%) reported that practicing OD was considered taboo (Table 6).

**Table 6:** Socio-cultural characteristics of respondents on OD practice in Ararso District, Jarar Zone, Somali Region, Eastern Ethiopia, 2023 (*n* = 630).

**Variables**	**Category**	**Frequency**	**Percentage**
There is taboo for defecating in an enclosed place (latrine)	Yes	227	36.0
	No	403	64.0
There is a taboo against defecating menstruating girls in latrine	Yes	229	36.3
	No	401	63.7
Defecating in an open space affects the marital acceptance of community members	Yes	374	59.4
	No	256	40.6
There is a taboo associated with sharing the same latrine with a mother-in-law or father-in-law	Yes	285	45.2
	No	345	54.8
There is a taboo associated with sharing the same latrine with a son-in-law or daughter-in-law	Yes	278	44.1
	No	352	55.9
Overall response about taboo	Taboo	335	53.2
	Not taboo	295	46.8

### Environmental or latrine-related factors

The majority, 565 (89.7%), of the households had pit latrines. Almost half, 343 (54.4%) of the latrines, were built < 18 months before the study period. Only 240 (36.8%) families shared a latrine with others. Of these, 128 (55.2%) households shared a latrine with more than two other households. On the other hand, 118 (16.4%) of the latrines were completely reconstructed. Out of 632 households interviewed, 204 (32.4%), used open fields near their houses while the latrine was not in service. Notably, 377 (59.8%) respondents said that there was open space near their home. In all, 424 (67.3%) latrines had a fresh footpath leading to the toilet, and there was a splash of urine or water on the latrine's surface. Only 261 (58.6%) households received water for toilet usage, and 336 (53.3%) latrines were not clean. In 439 (69.7%) households, women were responsible for cleaning latrines, while 357 (56.7%) households always cleaned their latrines. Of the 630 latrines, 324 (51.4%) required maintenance. The majority of the latrines, 556 (88.3%), had superstructures. Furthermore, 357 (56.7%) of the latrines were located more than five meters away from the dwellings. In addition, 357 (56.7%) latrines lacked a cover for the squatting hole, while 359 (57%) had a handwashing facility near the latrine (Table 7).

**Table 7:** Environmental or latrine-related factors in Ararso District, Jarar Zone, Somali Region, Eastern Ethiopia, 2023 (*n* = 630).

**Variables**	**Category**	**Frequency**	**Percentage**
Types of latrine	Pit-latrine	565	89.7
	VIP latrine	65	10.3
Service year of the latrine	< 18 months	343	54.4
	≥18 months	287	45.6
Sharing latrines among households	Yes	232	36.8
	No	398	63.2
The number of households that shared the latrine	< 2	104	44.8
	≥2	128	55.2
Upgrade or reconstruct the latrine	No	424	67.3
	Upgraded	90	14.3
	Reconstructed	116	18.4
Place of defecation when the latrine is not inservice	Neighborhoods	232	53.2
	Open field	204	32.4
The presence of open space close to your house	Yes	377	59.8
	No	253	40.2
Fresh foot path leading to the latrine and splash of urine or water on the latrine slab	Yes	424	67.3
	No	206	32.7
Availability of water for toilet use	Yes	261	41.4
	No	369	58.6
Latrine cleanliness status	Clean	308	48.9
	Unclean	322	51.1
Responsibility for cleaning the latrine in your family	Men	107	17.0
	Women	439	69.7
	Children	84	13.3
Need for latrine maintenance	Need maintenance	324	51.4
	Not need maintenance	306	48.6
Frequency of latrine cleaning	Always	357	56.7
	Sometimes	181	28.7
	Rarely	92	14.6
The presence of a latrine superstructure	No	74	11.7
	Yes	556	88.3
Latrine has good lighting	Yes	165	26.2
	No	465	73.8
Latrine hygienically separates human excreta from human contact	Yes	367	58.3
	No	263	41.7
Distance of the latrine from the house	< 5	273	43.3
	≥5	357	56.7
Latrine has cover on the squatting hole	Yes	273	43.3
	No	357	56.7
The presence of a handwashing facility near latrine	Yes	271	43.0
	No	359	57.0

**Figure 3:**
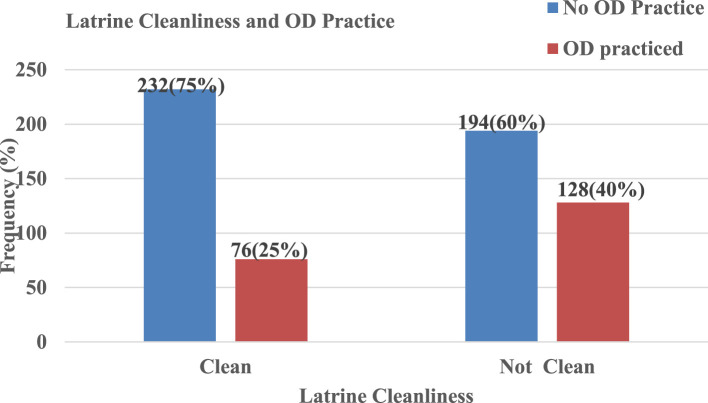
Open defecation practice and cleanliness of latrine in Ararso District, Jarar Zone, Ethiopia, 2023.

A three-fourth of HHs with clean latrines did not practice OD, whereas 40% of HHs with unclean latrines practiced OD (Figure 3).

### Factors associated with open defecation practices among households with latrines

In multivariable logistic regression, sex of respondents, educational status of household head, family size, educational status of household head, the presence of under-5-year-old children in the house, need for latrine maintenance, and latrine cleanliness status were significantly associated with open defection practice at *p* ≤ 0.05.

Accordingly, being men increased the likelihood of open defecation practice by 60% as compared to females (AOR = 1.60, 95% CI: 1.06, 2.4). The odds of open defecation practice among household heads who were illiterate were 3.5 times (AOR = 3.482; 95% CI: 2.426, 4.997) higher than literate household heads. The odds of open defecation practice increased by 62% among households with more than six members as compared to households with less than six members.

(AOR = 1.62, 95% CI: 1.09, 2.38). The odds of open defecation practice were 1.84 times higher among households with under-5-year-old children as compared to households without under-5-year-old children (AOR = 1.84, 95% CI: 1.22, 2.78). The odds of open defecation practice were 2.37 times (AOR = 2.37, 95% CI: 1.62, 3.48) higher among households with latrines that needed maintenance as compared to households that did not need maintenance. Furthermore, the odds of open defecation practice were 1.91 times (AOR = 1.91, 95% CI: 1.30, 2.81) higher among households with unclean latrines than those with clean latrines (Table 8).

**Table 8:** Factors associated with open defecation practice among HHs with latrines in multivariable logistic regression analysis in Ararso District, Jarar Zone, Somali Region, Ethiopia, 2023 (*n* = 630).

**Variable**	**Category**	**OD practice**		**AOR (95% CI)**
		**Yes (%)**	**No (%)**	
Sex of household head	Male	77 (39)	120 (61)	1.6 (1.06, 2.4)
	Female	127 (29)	306 (71)	1
Educational status of the household head	Illiterate	97 (52.4)	88 (47.6)	3.482 (2.426, 4.997)
	Litrate	107 (24)	338 (76)	1
Family size	≤ 6	82 (27)	226 (73)	1
	>6	122 (38)	200 (62)	1.62 (1.09, 2.38)
The presence of under-5-year-old children in the house	Yes	151 (38)	250 (62)	1.84 (1.22, 2.78)
	No	53 (23)	176 (77)	1
Latrines' need for maintenance	Need maintenance	134 (41)	190 (59)	2.37 (1.62, 3.48)
	Not need maintenance	70 (23)	236 (77)	1
Latrine cleanliness status	Clean	76 (25)	232 (75)	1
	Unclean	128 (40)	194 (60)	1.91 (1.30, 2.81)

## Discussion

The current study revealed that 32.4% of the rural households with latrines practiced open defecation. The findings of this study were slightly greater than those of the studies conducted in Machakle District, Northwest Ethiopia (27.8%), Wondo Genet District (16%), Raipur District, India (23.3%), and Kurnool District, India (27.6%) (2, 6, 19, 20). On the other hand, the prevalence of open defecation in this study was lower than in studies conducted in Southwest Ethiopia, Aneded District, Northwest Ethiopia, and South India, where 64.1%, 37%, and 54.8% of households with latrines practiced open defecation, respectively (4, 20, 21). The variations in the prevalence of open defecation practice among households with latrines might be due to awareness about latrine utilization among communities, socio-demographic characteristics within communities, differences in the sample size, and the year of the study. However, the finding of this study was consistent with a study conducted in rural districts of India, where 31% of households with latrines practiced open defecation (4).

Community latrine utilization is influenced by local culture and traditional beliefs. In the study area, 53.2% of respondents said that the presence of taboos in the communities discourages people from defecating or urinating in latrines, 36% had negative beliefs about toilet use, and 48.6% had a negative attitude toward latrine use. The findings were backed by a study conducted in Wonago District, southern nations, Ethiopia, and Borena, Ethiopia (22, 23). Cultural taboos, superstitions, attitudes, and religious beliefs can all have an impact on the acceptance and continuous use of improved latrines in rural areas. In rural areas, some tribes link latrines with impurity or badness, and they avoid defecating and urinating in latrines. In certain rural areas, menstrual women are not permitted to use the same latrine as men, and sharing a toilet between men and women is considered an obscenity (24). When questioned further, rural communities discovered that poor menstruation hygiene by women in rural communities is the cause of this condition (24).

In this study, the odds of open defecation practice were 1.60 times higher among men as compared to women, and the finding was in line with the study conducted in Chhattisgarh, India (25, 26). In Ethiopia, men engaged more in outdoor activities than females. Furthermore, the culture of the community encourages men to stay outside, far from home, for various outdoor activities such as farming, which does not allow them to be around the house where there are latrines. On the other hand, the culture and tradition prevent women from practicing open defecation because the community considers a lot of the women's behavior that is evaluated for marriage and other socially important conditions. That is why women are highly guarding their privacy and reputation.

In this study, the level of education was significantly associated with open defecation practice. The odds of open defecation (OD) were 3.482 times higher among household heads who were illiterate compared to literate household heads. This result was consistent with the study conducted in Chencha District, Southern Ethiopia, Chiro Zuria District, and West Harerghe Zone, Ethiopia (26, 27). This might be due to educated households having greater access to sanitation and hygiene information than illiterate households. As a result, educated households used their latrine facilities more than illiterate households.

Family size was significantly associated with open defecation practices. The odds of open defecation practice were 1.68 times higher among family sizes greater than six compared to family sizes less than six. This finding was in line with a study conducted in Addis Ababa (28). The reason might be that households with more family members lead to queuing for the latrine, and anyone who cannot wait for the toilet has a higher tendency to practice open defecation. Furthermore, higher family members reduced the cleanliness of the toilet, and when the toilet became dirty, individuals were forced to defecate outside.

The presence of under-5-year-old children within households was significantly associated with open defecation practices. The odds of open defecation (OD) practice were two times higher among households with under−5-year-old children compared to those households without under-5-year-old children. This result was in line with the studies done in Dembia District, Wondo Genet District, southwest Ethiopia, and eastern Nepal (6, 20, 29). The possible justification was that under-5-year-old children cannot use the toilet, and when the urge to defecate comes, they defecate openly if there is no one around to give them popo. Also, parents do not emphasize the open defecation of the children, but rather adults.

Latrines' need for maintenance was significantly associated with open defecation practices. The odds of open defecation (OD) practice were 2.34 times higher among households having latrines that required maintenance as compared to those households' latrines that did not need latrine maintenance. This result was in agreement with the study conducted in Wondo Genet and southwest Ethiopia (6, 20). This might be due to households with unmaintained latrines reflecting various problems such as leakage, privacy issues, and a lack of comfort that may hinder their use. Also, unmaintained and deteriorated latrines will expose them to different accidents, like falling. These conditions pushed some household members to defecate in the open, especially where there are opportunities for them to practice open defecation.

Furthermore, the practice of open defecation in the study area was significantly associated with the latrine cleanliness status. The odds of open defecation practice were 1.96 times higher among households with unclean latrines compared to those with clean latrines. This study was in line with the studies done in Dembia District, Aneded District, North West Ethiopia, and Laelai Maichew Woreda, Aksum, Tigray, Ethiopia (21, 30). This might be due to the user's fear of using contaminated, breeding sites of fly, unclean, odorous, and dirty toilets. People prefer and are motivated to use a clean and attractive latrine. For this reason, individuals prefer defecating in an open environment rather than using a dirty toilet.

## Conclusion

Even though every household had a latrine, open defecation was common in the study area. In total, 32.4% of households with latrines experience open defecation in outdoor environments. The sex of the household head, family size, the presence of under-5-year-old children in the household, the need for latrine maintenance, latrine cleanliness status, and the educational status of the household head were significantly associated with open defecation practice. In conclusion, toilet coverage was high in the study area, but the presence of a latrine facility alone may not be sufficient to eliminate open defecation practice without addressing factors that encourage the practice. As a result, the Ararso District Health Bureau must give attention to the above-identified factors to reduce OD open defecation practices among households with latrines through appropriate design of sanitation and hygiene promotion. Health extension workers must closely follow the community found remote from health posts to undertake sanitation and hygiene education promotion that improves toilet utilization behavior, particularly among women who likely spend the majority of their time caring for their children.

## Data availability statement

The raw data supporting the conclusions of this article will be made available by the authors, without undue reservation.

## Ethics statement

To undertake this study, the Institutional Health Research Ethics Review Committee (IHRERC) of College of Health and Medical Sciences, Haramaya University provided ethical approval. The participants provided informed consent for their participation in the study.

## Author contributions

AI: Writing – original draft, Supervision, Project administration, Investigation, Formal analysis, Data curation, Conceptualization. MI: Formal analysis, Methodology, Writing – review & editing. MA: Formal analysis, Methodology, Software, Writing – review & editing. AG: Formal analysis, Investigation, Methodology, Validation, Writing – review & editing. YM: Formal analysis, Methodology, Software, Validation, Writing – review & editing. DA: Methodology, Software, Writing – original draft. AC: Methodology, Software, Writing – review & editing.

## References

[1] Saleem M Burdett T Heaslip V. Health and social impacts of open defecation on women: a systematic review. BMC Public Health. (2019) 19:1–12. 10.1186/s12889-019-6423-z 30727975 PMC6364430

[2] Abathun T. Open Defecation Practice and Associated factors Among Households who have A Latrine in Rural Communities of Machakl District, East Gojjam Zone, Amhara Region, Northwest, Ethiopia: A Community Based Cross Sectional Study. (2020).

[3] Coffey D Gupta A Hathi P Khurana N Srivastav N Vyas S. Open defecation: evidence from a new survey in rural north India. Econ Politi Weekly. 2014:43–55. Available online at: https://www.jstor.org/stable/2448070 38288391 PMC10824488

[4] Yogananth N Bhatnagar T. Prevalence of open defecation among households with toilets and associated factors in rural south India: an analytical cross-sectional study. Trans R Soc Trop Med Hyg. (2018) 112:349–60. 10.1093/trstmh/try064 30032253

[5] Bathija GV Sarvar R. Defecation practices in residents of urban slums and rural areas of hubballi, Dharwad: a cross sectional study. Int J Community Med Public Health. (2017) 4:724–8. 10.18203/2394-6040.ijcmph20170747

[6] Ashenafi T Dadi AF Gizaw Z. Latrine utilization and associated factors among Kebeles declared open defecation free in Wondo Genet district, South Ethiopia, 2015. ISABB J Health Environm Sci. (2018) 5:43–51 10.5897/ISAAB-JHE2018.005038147025

[7] Sinha A Nagel CL Thomas E Schmidt WP Torondel B Boisson S Clasen TF. Assessing latrine use in rural India: a cross-sectional study comparing reported use and passive latrine use monitors. Am J Tropical Med Hyg. (2016) 95:720. 10.4269/ajtmh.16-0102 27458042 PMC5014284

[8] Jenkins MW Scott B. Behavioral indicators of household decision-making and demand for sanitation and potential gains from social marketing in Ghana. J Social science medicine (Baltimore). (2007) 64:2427–42. 10.1016/j.socscimed.2007.03.010 17442472

[9] Tilley E. Compendium of Sanitation Systems and Technologies: Eawag. (2014).31294713

[10] MacGill M. What You Should Know About Diarrhea. (2023). Available online at: https://wwwmedicalnewstodaycom/ (accessed August 31, 2023).

[11] Fenta A Alemu K Angaw DA. Prevalence and associated factors of acute diarrhea among under-five children in Kamashi district, western Ethiopia: community-based study. BMC Pediatr. (2020) 20:1–7. 10.1186/s12887-020-02138-1 32429989 PMC7236964

[12] Suthar P Joshi NK Joshi V. Study on the perception of Swachh Bharat Abhiyan and attitude towards cleanliness among the residents of urban Jodhpur. J family Med Prim Care. (2019) 8:3136–9. 10.4103/jfmpc.jfmpc_502_19 31742132 PMC6857413

[13] Patil SR Arnold BF Salvatore AL Briceno B Ganguly S Colford JM. Jr.. The effect of India's total sanitation campaign on defecation behaviors and child health in rural Madhya Pradesh: a cluster randomized controlled trial. PLoS Med. (2014) 11:e1001709. 10.1371/journal.pmed.1001709 25157929 PMC4144850

[14] Coffey D Gupta A Hathi P Khurana N Spears D Srivastav N. Revealed Preference for Open Defecation: evidence froma new survey in rural North India. Econ Political Weekly. (2014) 49:43–55. Available online at: https://www.jstor.org/stable/24480705 PMC10824488 38288391

[15] Temesgen A Molla Adane M Birara A Shibabaw T. Having a latrine facility is not a guarantee for eliminating open defecation owing to socio-demographic and environmental factors: the case of Machakel district in Ethiopia. PLoS ONE. (2021) 16:e0257813. 10.1371/journal.pone.0257813 34591873 PMC8483416

[16] Supply WUJW Programme SM. Progress on drinking water and sanitation: 2014 Update. Geneva: World Health Organization. (2014).

[17] Lemma T Abera K Sintayehu BH Hailu FD Mesfin TS. Latrine utilization and associated factors among kebeles implementing and non implementing urban community led total sanitation and hygiene in Hawassa town, Ethiopia. African J Environm Sci Technol. (2017) 11:151–62. 10.5897/AJEST2016.222338147025

[18] Banerjee AB Pasha M Fatima A Isaac A. Study of open air defecation practice in rural Nandivargam village. J Space. (2013) 70:90. Available online at: https://www.academia.edu/33136938/A_study_of_open

[19] Panda PS Chandrakar A Soni GP. Prevalence of open air defecation and awareness and practices of sanitary latrine usage in a rural village of Raipur district. Int J Comm Med Public Health. (2017) 4:3279–82. 10.18203/2394-6040.ijcmph20173828

[20] Oljira D Berkessa T. Latrine use and determinant factors in Southwest Ethiopia. J Epidemiol Public Health Rev. (2016) 1:1–5. 10.16966/2471-8211.133 PMC4736163 26830027

[21] Chanie T Gedefaw M Ketema K. Latrine utilization and associated factors in rural community of Aneded district, North West Ethiopia, 2014. J Community Med Health Educ. (2016) 6:1–12. 10.4172/2161-0711.1000478

[22] Tamene A Afework A. Exploring barriers to the adoption and utilization of improved latrine facilities in rural Ethiopia: an integrated behavioral model for water, sanitation and hygiene (IBM-WASH) approach. PLoS ONE. (2021) 16:e0245289. 10.1371/journal.pone.0245289 33428677 PMC7799797

[23] Mosisa M Mesele A Bogale B Work K. Sanitation in Borena pastoral community of Ethiopia: Pinpointing the status and challenges. Ethiopian J Sci Sustain Dev. (2019) 6:1. Available online at: https://www.academia.edu/40775915/Sanitation_in

[24] Ferdinandes A. Understanding pastoralists and their water, sanitation and hygiene needs. In: Discussion Paper Water Aid. Tanzania: IRC (2011).

[25] Kawale SK Thakur H Sharma V Minz A. Socio-demographic factors affecting utilization of toilet among peoples attending tertiary care hospital at Bilaspur, Chhattisgarh. Int J Commun Med Public Health. (2018) 5:1167–71. 10.18203/2394-6040.ijcmph20180778

[26] Dagnew GG Abebaw AF Wake SL Derso AG. Assessment of latrine use and associated factors among rural community members in Chiro Zuria Woreda particularly in Kilinso and Nejebas Kebele. J Microb Biochem Technol. (2019) 11:2430.

[27] Koyra HC Sorato MM Unasho YS Kanche ZZ. Latrine utilization and associated factors in rural Community of Chencha District, southern Ethiopia: a community based cross-sectional study. Am J Public Health Res. (2017) 5:98–104. 10.12691/ajphr-5-4-237223222

[28] Adane M Mengistie B Kloos H Medhin G Mulat W. Sanitation facilities, hygienic conditions, and prevalence of acute diarrhea among under-five children in slums of Addis Ababa, Ethiopia: Baseline survey of a longitudinal study. PLoS ONE. (2017) 12:e0182783. 10.1371/journal.pone.0182783 28854200 PMC5576656

[29] Budhathoki SS Shrestha G Bhattachan M Singh SB Jha N Pokharel PK. Latrine coverage and its utilisation in a rural village of Eastern Nepal: a community-based cross-sectional study. BMC Res Notes. (2017) 10:1–7. 10.1186/s13104-017-2539-3 28606171 PMC5469064

[30] Gebremedhin G Tetemke D Gebremedhin M Kahsay G Zelalem H Syum H. Factors associated with latrine utilization among model and non-model families in Laelai Maichew Woreda, Aksum, Tigray, Ethiopia: comparative community based study. BMC Res Notes. (2018) 11:1–7. 10.1186/s13104-018-3683-0 30103799 PMC6090671

